# Primary Endometrial Adenocarcinoma with Signet-Ring Cells: A Rarely Observed Case and Review of the Literature

**DOI:** 10.1155/2015/404692

**Published:** 2015-11-23

**Authors:** Aslı Kahraman Akkalp, Eser Sefik Ozyurek, Umit Seza Tetikkurt, Senay Yalcin, Yazgi Koy, Abdullah Taner Usta

**Affiliations:** ^1^Pathology Department, Bagcilar Research and Training Hospital-Istanbul, Merkez Mahallesi Esenler, Caddesi 6, Sokak No. 1, Bagcilar, Istanbul, Turkey; ^2^Gynecology and Obstetrics Department, Research and Training Hospital-Istanbul, Merkez Mahallesi Esenler, Caddesi 6, Sokak No. 1, Bagcilar, Istanbul, Turkey

## Abstract

An extremely rare case of a “primary endometrial adenocarcinoma with signet-ring cells” is presented in this study with microscopical images of the characteristic coexistence of the tumour and intermediate precancerous areas containing signet-ring cells.

## 1. Introduction

Presence of signet-ring cells in primary endometrial adenocarcinoma tumours is a rarely observed entity which has to our knowledge been reported in only 5 cases, previously [1–4] (Table 1).

When signet-ring cells are observed in uterine tumours, the probability of these tumours being metastatic from nongenital organs is relatively higher. In these tumours, the probability of metastasis from the gastrointestinal system or the breast has to be ruled out. In the form accompanying primary endometrial adenocarcinoma tumours, signet-ring cells are neighbored by endometrial carcinoma and endometrial hyperplasia foci.

## 2. Case Presentation

**Figure 1:**
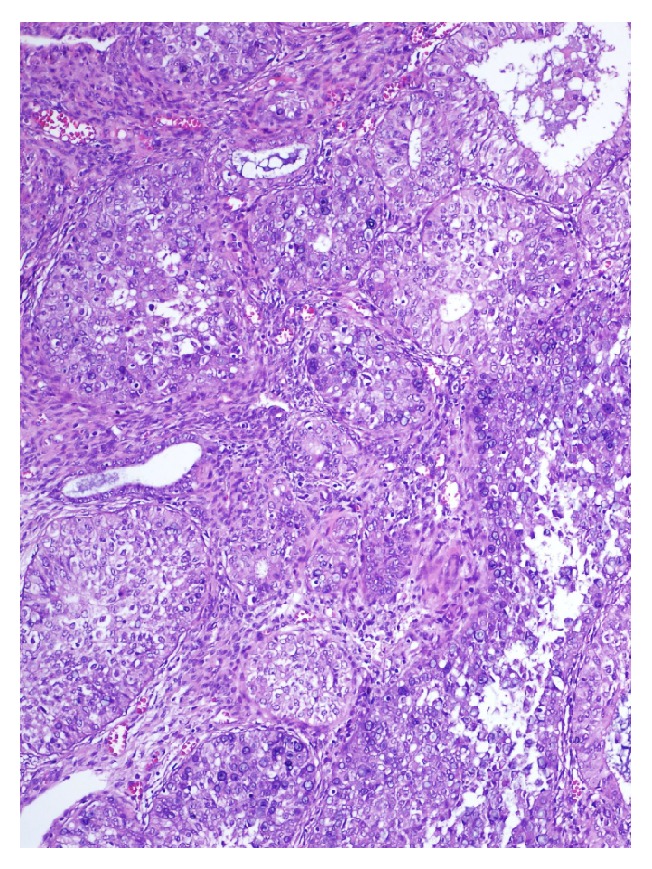
Low grade carcinoma areas and signet-ring cells; H&E ×110.

**Figure 2:**
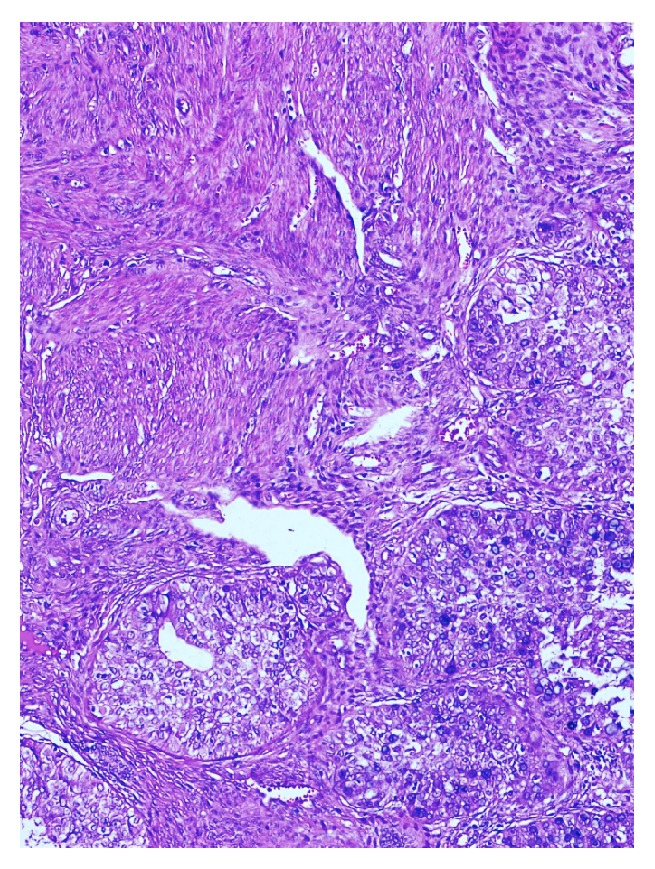
Tumour cells in signet-ring cell morphology; H&E ×220.

**Figure 3:**
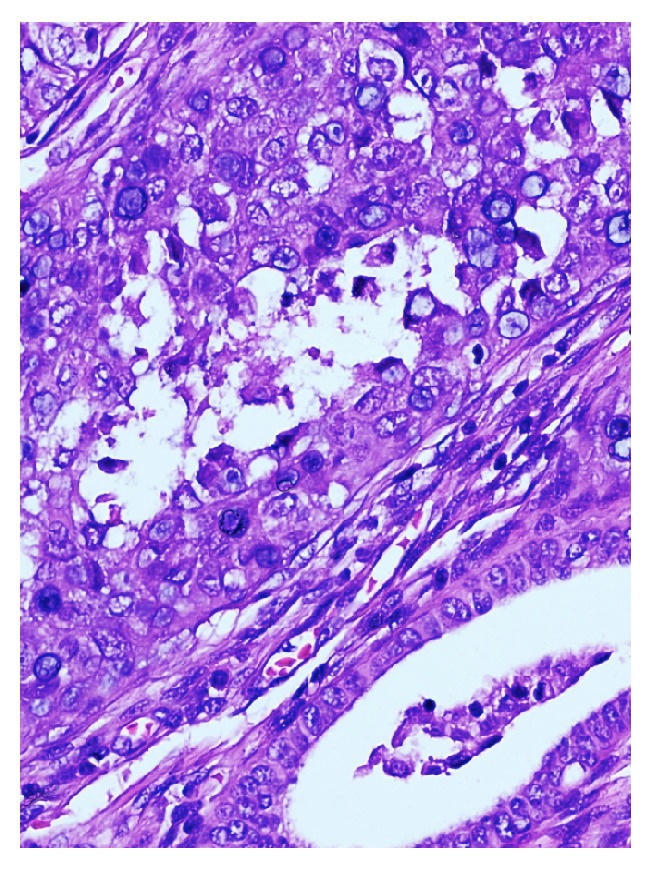
Hyperplastic endometrial gland structures and tumour cells with signet-ring cell morphology; H&E ×440.

**Figure 4:**
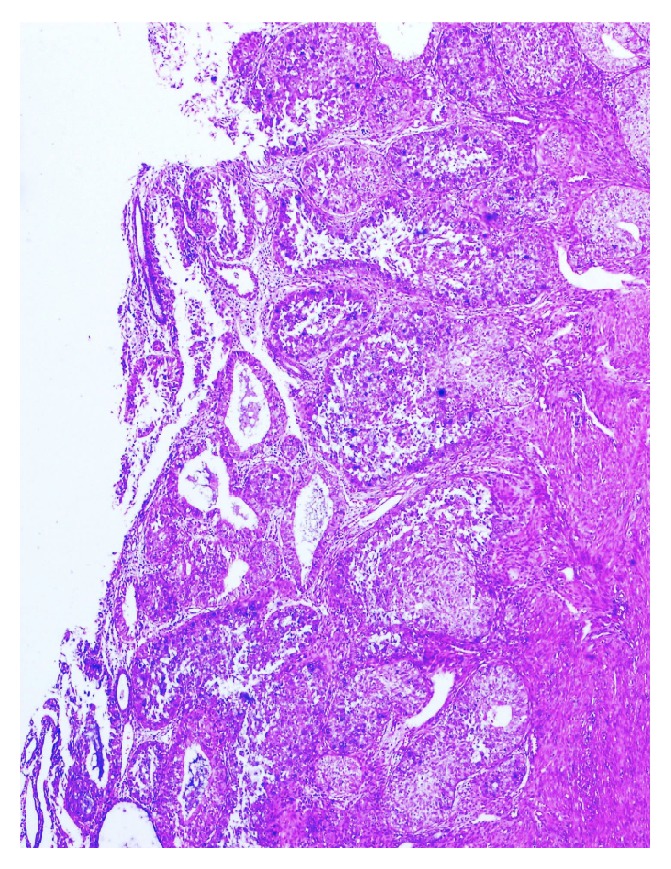
Superficial tumoral invasion; H&E ×110.

Our case was a 77-year-old woman who presented to the obstetrics and gynecology outpatient section of the Bagcilar Research and Training Hospital, Istanbul, with postmenopausal bleeding. Endometrial sampling was performed and the specimen was sent for examination in our department of pathology. Histopathological examination revealed the extensive dissipation of signet-ring cells within endometrial adenocarcinoma areas composed of stromal and glandular components (Figures 1, 2, and 3). Limited myometrial invasion (less than 50%) was observed in the resection material (Figure 4).

**Figure 5:**
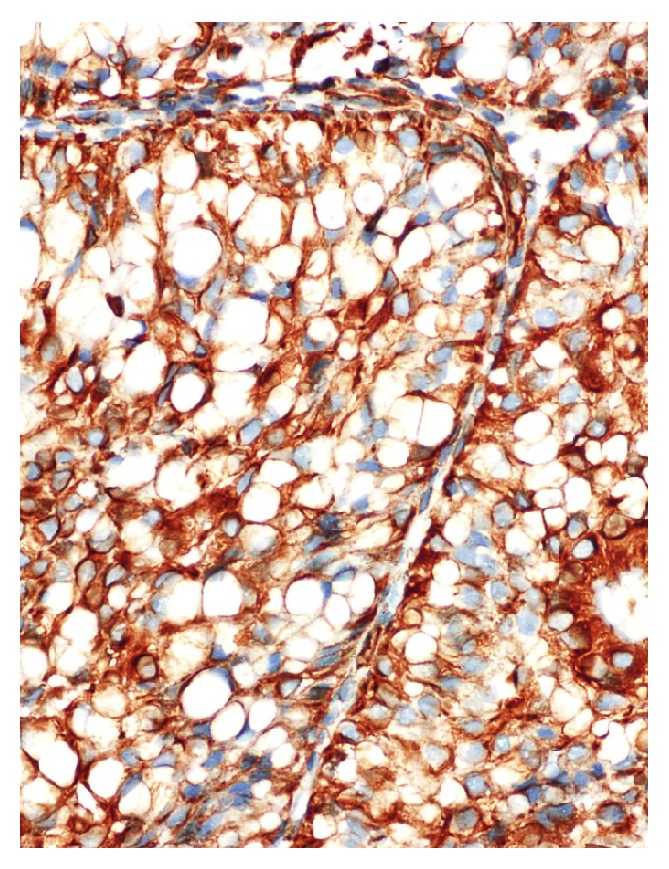
Cytoplasmic staining with vimentin; (H&E) ×440.

**Figure 6:**
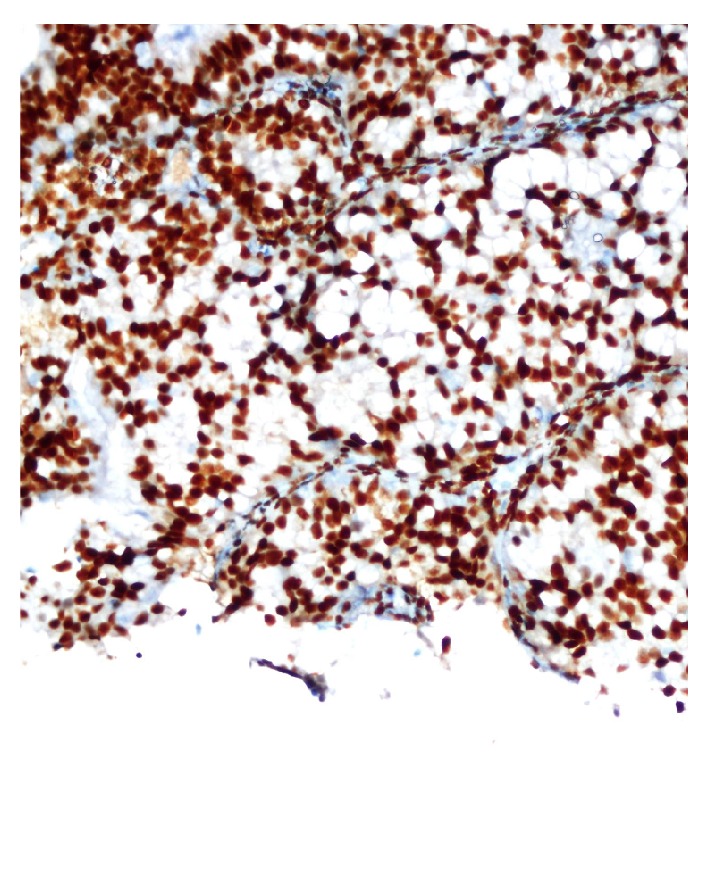
Positive staining for estrogen receptors.

**Figure 7:**
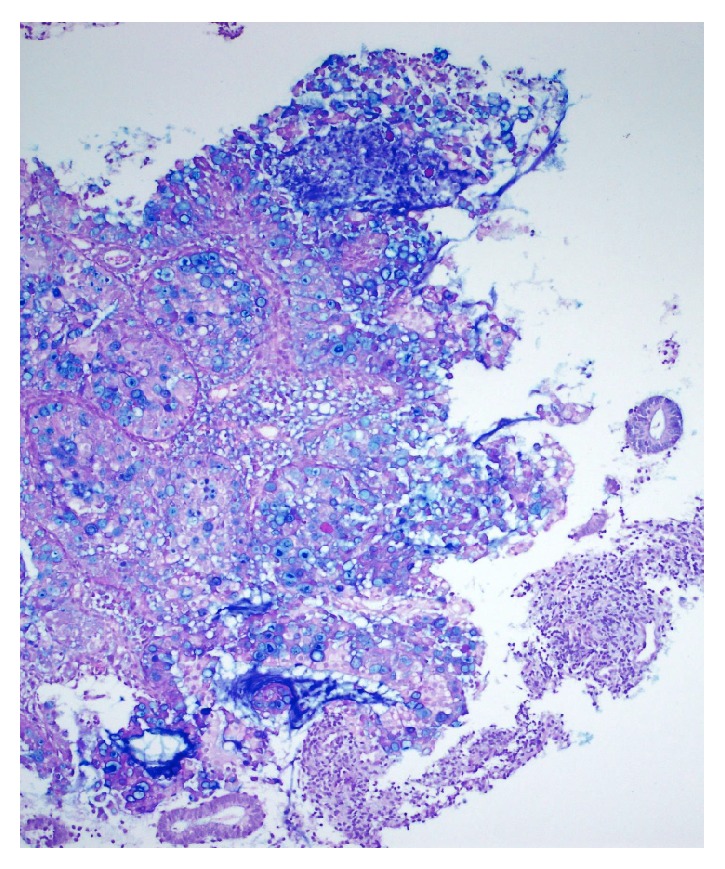
Intracytoplasmic mucin deposition visualized with Alcian Blue.

Immunohistochemical studies revealed positive staining for cytokeratin 7 (CK7), vimentin, and estrogen receptors (ER) (Figures 5 and 6), whereas no staining for CEA, CDX2, Gross Cystic Disease Fluid Protein-15 (GCDFP-15), or cytokeratin 20 (CK20). With the additional application of the Alcian Blue and Mucicarmin stains, the presence of mucin was defined within the signet-ring cells (Figure 7). Radiological and endoscopic evaluations did not reveal the presence of any other tumour. Accounting for the histopathology and the immunohistochemistry, the case was reported as “endometrial adenocarcinoma with signet-ring cells.” In examining the subsequent TLH + BSO specimen, the tumour surrounded by areas of simple/complex hyperplasia with atypia similar to the histology of the curettage material was defined. The tumour had infiltrated less than the inner half of the myometrium; the rest was normal. This latter specimen was also reported as “endometrial adenocarcinoma with signet-ring cells FIGO grade 2.” Postoperative external beam radiotherapy was added and at the time of reporting there is no evidence of recurrence.

## 3. Discussion

Signet-ring cells are vacuolated cells in which the nucleus is displaced and the vacuolated cytoplasm forms the image of a finger pore. The presence of signet cells in the presence of an endometrial tumour is primarily considered as the sign of a metastatic tumour [5]. Signet-ring cell adenocarcinomas of the endometrium are extremely rare tumours. While the World Health Organization (WHO) classification lists the pure form of the carcinoma with signet-ring cells as a rare cervical histological variant, it is not listed among the histological tumour variants of the uterine corpus [6, 7].

To our knowledge, only 5 cases of  “endometrial adenocarcinoma containing signet-ring cells” have been reported so far in the literature (Table 1). Pusiol et al. have suggested that the histopathological image of endometrial adenocarcinoma and hyperplasia accompanied by signet-ring cells should be referred to as endometrial adenocarcinoma containing signet-ring cells, whereas the term signet-ring cell endometrial adenocarcinoma should be reserved only for cases where the whole tumour is comprised of signet-ring cells. Similarly, in previous publications and in our case, endometrial adenocarcinoma and hyperplasia are accompanied by signet-ring cells of various proportions.

When a carcinoma of the uterine corpus is histopathologically accompanied by signet cells, breast, stomach, or colon should should be kept in mind as possible origins of primary tumours. Findings which rather suggest a metastatic tumour are diffuse stromal infiltration, absence of precursor components, and substantial lymphovascular-space invasion. In our case, neither the whole body screening nor the gastroscopy/colonoscopy evaluations revealed an extrapelvic primary tumour.

Immunohistochemistry, as well, confirmed a primary endometrial tumour with negativity for GCDFP-15, CEA, CDX2, CK20; and positivity for CK7, ER, and Vimentin.

## 4. Conclusion

This extremely rare case of an “endometrial adenocarcinoma with signet-ring cells” with an interesting histopathological view is, to our knowledge, the sixth reported case in literature. The identification of a “primary endometrial adenocarcinoma with signet-ring cells” requires ruling out metastatic tumours as well as identifying an endometrial origin with the histopathology and immunohistochemical characteristics [1]. Both of these steps are essential undertakings because the presence of a metastatic tumour presenting with a signet-ring cell positivity most probably notes a breast or a gastric primary tumour, an advanced stage disease, and a different therapeutical approach. On the other hand, considering the limited clinical data, we have, as of now, primary endometrial adenocarcinoma with signet-ring cells that has not shown a favorable prognosis in all cases.

## Figures and Tables

**Table 1:** Endometrial adenocarcinoma with signet-ring cells: review of the literature.

Case	Authors	Age/years	Diagnosis	Treatment	Stage	Followup
1	Mooney et al., 1997 [2]	65	SRC^*∗∗∗*^	Hysterectomy, BSO^*∗*^, and pelvic and paraaortic LND^*∗∗*^. Partial O. abdominal, pelvic washing	NS	Free of disease 6 months after surgery

2	Chebib et al., 2010 [3]	51	Primary SRC	Hysterectomy, BSO, and pelvic LND. Abdominal, pelvic washing	FIGO IVB	Death of metastatic disease 6 months after surgery

3	Boyd et al., 2010 [4]	46	Primary mucinous adenocarcinoma of the endometrium with signet-ring cells arising in adenomyosis	Subtotal hysterectomy	NS	NS

4	Boyd et al., 2010 [4]	59	Primary endometrioid adenocarcinoma of the endometrium with signet-ring cells	Hysterectomy	NS	NS

5	Pusiol, 2014 [1]	53	HPV11 Positive Endometrioid Carcinoma of the Endometrium with Signet-Ring Cells	Hysterectomy, BSO, pelvic and paraaortic LND	FIGO stage IB	Free of disease 22 months after surgery

Case presented		53	Primary endometrioid adenocarcinoma of the endometrium with signet-ring cells	Radical hysterectomy with BSO and pelvic LND	FIGO stage IA	Free of disease 14 months after surgery

^*∗*^Bilateral salpingooopherectomy.

^*∗∗*^Lymph node dissection.

^*∗∗∗*^Signet-ring cells.
